# Real-world effects of antidepressants for depressive disorder in primary care: population-based cohort study

**DOI:** 10.1192/bjp.2024.194

**Published:** 2025-05

**Authors:** Franco De Crescenzo, Riccardo De Giorgi, Cesar Garriga, Qiang Liu, Seena Fazel, Orestis Efthimiou, Julia Hippisley-Cox, Andrea Cipriani

**Affiliations:** Department of Psychiatry, University of Oxford, UK; Oxford Health National Health Service (NHS) Foundation Trust, Warneford Hospital, Oxford, UK; Nuffield Department of Primary Care Health Sciences, University of Oxford, UK; Department of Engineering Mathematics and Technology, University of Bristol, UK; Institute of Social and Preventive Medicine (ISPM), University of Bern, Switzerland; Institute of Primary Health Care (BIHAM), University of Bern, Switzerland; Oxford Precision Psychiatry Lab, National Institute for Health and Care Research (NIHR) Oxford Health Biomedical Research Centre, Oxford, UK; Department of Psychiatry, University of Oxford, UK; and Oxford Health National Health Service (NHS) Foundation Trust, Warneford Hospital, Oxford, UK; Department of Psychiatry, University of Oxford, UK; Oxford Health National Health Service (NHS) Foundation Trust, Warneford Hospital, Oxford, UK; and Oxford Precision Psychiatry Lab, National Institute for Health and Care Research (NIHR) Oxford Health Biomedical Research Centre, Oxford, UK

**Keywords:** Antidepressants, depressive disorders, register-based epidemiology, general adult psychiatry, observational study

## Abstract

**Background:**

Antidepressants’ effects are established in randomised controlled trials (RCTs), but not in the real world.

**Aims:**

To investigate real-world comparative effects of antidepressants for depression and compare them with RCTs.

**Method:**

We performed a cohort study based on the QResearch database. We included people with a newly recorded diagnosis of depression, exposed to licensed antidepressants in the UK. We assessed all-cause dropouts (acceptability), dropouts for adverse events (tolerability), occurrence of at least one adverse event (safety), and response and remission on the Patient Health Questionnaire (PHQ)-9 (effectiveness) at 2 and 12 months. Logistic regressions were used to compute adjusted-odds ratio (aOR) with 99% CIs, assessing the associations between exposure to each antidepressant against fluoxetine (comparator) and outcomes of interest. We compared estimates from the real world with RCTs using ratio-of-odds ratio (ROR) with 95% CI.

**Results:**

A total of 673 177 depressed people were studied: females 57.1%, mean age 42.8 (s.d. 17.7) years, mean baseline PHQ-9 17.1 (s.d. 5.0) (moderately severe depression). At 2 months, antidepressant acceptability was 61.4%, tolerability 94.4%, safety 54.5%, PHQ-9 decreased to 12.3 (s.d. 6.5). At 12 months, acceptability was 12.3%, tolerability 87.5%, safety 28.8%, PHQ-9 12.9 (s.d. 6.8). In the short and long term, tricyclics, mirtazapine and trazodone were worse than fluoxetine for most outcomes; citalopram had better acceptability than fluoxetine (aOR 0.95; 99% CI 0.92, 0.97), sertraline had lower tolerability (aOR 1.12; 99% CI 1.06, 1.18), and both citalopram and sertraline had lower safety (aOR 1.17 and 1.25, respectively). In the long term, citalopram had better acceptability (aOR 0.78; 99% CI 0.76, 0.81) and effectiveness (aOR 1.12 for both response and remission), but worse tolerability (aOR 1.09; 99% CI 1.06, 1.13) and safety (aOR 1.12; 99% CI 1.08, 1.16). Observational and randomised data were similar for citalopram and sertraline, while there was some difference for drugs less prescribed in the real world.

**Conclusions:**

Antidepressants showed low acceptability, moderate-to-high tolerability and safety, and small-to-moderate effectiveness in the real world. Real-world and RCT estimates showed similar findings only when the analyses were carried out using large datasets; otherwise, the results diverged.

Depression affects more than 300 million people worldwide.^1^ Evidence supports the use of both pharmacological and psychological therapies,^2,3^ but guidelines recommend initiating antidepressant medications for adults with moderate to severe depression.^2–7^ Antidepressants are commonly used: in England alone, 79.4 million antidepressant prescriptions were issued to 7.87 million people in 2020–2021.^8^

In randomised controlled trials (RCTs), 26% of people on average discontinue antidepressants for any reason, including inefficacy (i.e. acceptability), while approximately 10% stop them for side effects (i.e. safety) that cannot be tolerated (i.e. tolerability) after 2 months of treatment.^9^ Of those who continue taking antidepressants, only about 50% respond to first-line treatment (i.e. efficacy).^10^ However, RCTs often exclude individuals with concurrent illness and medication use, despite the high prevalence of multimorbidity (68%) and polypharmacy (63–98%) in those who will subsequently require treatment outside the trial setting (i.e. real-world patients).^11^ Exclusion of these large populations may limit the generalisability and transportability of RCT findings to clinical practice.^12^ This may contribute to an ‘efficacy-effectiveness gap’, where efficacy relates to the performance of drugs in RCTs, and effectiveness to real-world settings.^13^ Recently, real-world and RCT data on the effectiveness of antipsychotics for schizophrenia have shown high congruency.^14^ It is currently unclear, however, whether the same holds for antidepressants in people with depression.

This study aims to assess the overall and comparative acceptability, tolerability, safety and effectiveness of antidepressants in a large, representative sample of adults diagnosed with depressive disorders. Furthermore, we compare real-world estimates with pooled, summary-level data available from equivalent RCTs,^9^ to assess the degree of agreement between the two sources of evidence, and the existence of an efficacy-effectiveness gap in antidepressants.

## Method

The study methods are reported in full in the published protocol.^15^

### Population

We extracted an open cohort of individuals registered for at least 12 months with eligible general practices between 1 January 1998 and 15 August 2020 from the QResearch primary care research registry, version 45. QResearch is a large, consolidated database derived from the anonymised health records from general practices using the Egton Medical Information Systems (EMIS) of over 35 million people in England (www.QResearch.org). We included people aged 18–100 years who had a newly recorded diagnosis of a depressive disorder according to their ‘Read codes’, using case definitions^15^ that have been applied in earlier studies.^16,17^ Participants included those who had been started on any licensed antidepressant monotherapy and were followed up for 12 months.

We excluded people with a diagnosis of bipolar disorder or schizophrenia spectrum disorder; those with a diagnosis of postpartum depression made within 180 days before or up to 180 days after the first diagnosis of depression; those with more than one antidepressant prescribed at baseline; those who took antidepressants more than 60 days before the diagnosis of depression; those with an antipsychotic or mood stabiliser prescription; and those with a previous antidepressant prescription or diagnosis of depression before the cohort entry date.

The final cohort for analysis included an exposed group of participants on each antidepressant monotherapy.

### Drug exposure

The exposure of interest was the prescription of any one of 30 antidepressants licensed in the UK – from the full list previously published.^15^ Fluoxetine was utilised as the index comparator, as this drug was the first selective serotonin reuptake inhibitor (SSRI) approved by regulatory agencies internationally and it has been the most used active comparator in antidepressant clinical trials.^9^

Information was extracted from all prescriptions of antidepressants issued during the 12-month follow-up according to previously validated criteria.^16,17^ The duration of each prescription was calculated by dividing the number of tablets prescribed by the number of tablets to be taken each day. If information on ‘tablets per day’ was missing or not sufficiently detailed, we estimated the duration of the prescription considering the most used prescription, i.e. once daily. Participants were considered as continually exposed to an antidepressant for periods with no gaps of more than 30 days between the end of one prescription and the beginning of the next.

### Outcomes

According to the protocol, the outcomes of interest were measured at 2 months (i.e. short term) and 12 months (i.e. long term) after the initial prescription of antidepressants, as follows:

- Acceptability of treatment was measured as the proportion of people who dropped out because of any cause. We defined treatment dropout as follows:^15^ (i) a person had a gap of more than 30 days between the end of a prescription of an antidepressant and the start of the next prescription; (ii) a person switched to another antidepressant (i.e. switch strategy); (iii) a person was prescribed an additional antidepressant (i.e. combination strategy); (iv) a person was prescribed a mood stabiliser or an antipsychotic (i.e. augmentation strategy).
- Tolerability of treatment was measured as the proportion of people who dropped out because of any adverse event.
- Safety of treatment was measured as the proportion of people with at least one adverse event, irrespective of whether they dropped out. We selected ‘Read codes’ for 67 adverse events that have been identified in clinical trials as severe and frequently associated with antidepressants,^18^ or shown to be important to patients, carers and healthcare professionals in a recent large survey.^19^
- To compare real-world estimates with outcome data from RCTs, effectiveness of treatment at the timepoint closest to 2 and 12 months was measured as response (i.e. 50% reduction on the Patient Health Questionnaire (PHQ)-9^20^) and remission (i.e. scoring less than 5 on the PHQ-9), by dichotomising the PHQ-9 scores using validated methods. Other depression scales were transformed to PHQ-9 scores, which range from 0 to 27, following Wahl et al.^21^ If PHQ-9 data were missing at follow-up, we used the PHQ-9 score closest to a specific follow-up timepoint within a predefined time window. This approach ensured that the most relevant data informed the analysis, while still allowing for flexibility in the timing of follow-up assessments. By using the value closest to the specified timepoint, we aimed to provide a more accurate representation of the individual's status at that follow-up period.

### Confounders

Based on previous studies of antidepressants in QResearch,^16,17^ we considered several confounding baseline variables, i.e. possible risk factors for the outcomes of interest, potentially also associated with the likelihood of receiving a particular antidepressant treatment. Suspected confounders included: gender, age, baseline severity, depression subtype, year of diagnosis, body mass index (BMI), smoking status, daily alcohol intake, ethnicity, Townsend quintile, regions of England, comorbidities and use of other drugs. See Table 4 for the detailed list of confounders.

### Statistical analyses

We described the study population by reporting baseline characteristics and antidepressant use. We used multiple imputation by chained equations to impute data when actual values were not available.^22^ For each imputation, we generated ten imputed datasets and combined coefficient estimates across these using Rubin's rule. All confounding and outcome variables were included in the multiple imputation process (see Supplementary Appendix 1 available at https://doi.org/10.1192/bjp.2024.194).

To estimate relative effects between antidepressant monotherapy and fluoxetine (index comparator), we used multivariable logistic regression models. These models were clustered by general practices and adjusted for all confounders. We used a separate model for each of the four outcomes, i.e. acceptability, tolerability, safety and effectiveness, and estimated adjusted-odds ratios (aOR) with 99% CIs (99% CIs). We used intention-to-treat (ITT) analyses, i.e. we analysed each person according to the drug they were prescribed, irrespective of whether they received it to the end. We excluded people who switched antidepressants prior to entering the study cohort. However, for those who switched antidepressants during follow-up, they were analysed as part of that treatment group regardless of subsequent changes in their medication. We visualised results using a Kilim plot, where rows indicated drugs, columns indicated outcomes and each cell showed effects versus the reference, for the corresponding outcome; cells were coloured according to *P*-values, to visualise the strength of statistical evidence against the null hypothesis of no difference versus the reference.^23^

To assess the robustness of our real-world observational estimates of relative (i.e. versus fluoxetine) treatment effects, we examined them in comparison with findings obtained from the largest network meta-analysis of RCTs to date (Group of Researchers Investigating Specific Efficacy of Individual Drugs for Acute depression: GRISELDA).^9^ We compared our estimates for all outcomes at 2 months (i.e. short-term) with estimates for acceptability, tolerability, response and remission available. We could not compare safety data as these were not published in GRISELDA. For this analysis, we used only the antidepressants that were studied in both databases, and we focused on the most frequently used antidepressants in QResearch for which we could develop a logistic regression model, i.e. amitriptyline, trazodone, citalopram, escitalopram, paroxetine, sertraline, duloxetine, mirtazapine and venlafaxine. We calculated for each drug the ratio-of-odds ratio (ROR) versus fluoxetine: real-world odds ratio of each drug versus fluoxetine, compared with RCTs odds ratio of the same drug versus fluoxetine, together with 95% CI Stata MP 16.0 software^24^ (StataCorp, Texas, USA; see https://www.stata.com/support/faqs/resources/citing-software-documentation-faqs/) was used for the statistical analyses.

### Institutional review board (IRB) approval

This project was independently peer-reviewed by the QResearch scientific committee and approved on 1 July 2019 with reference 18/EM/0400.

#### Registration

QResearch scientific committee reference 18/EM/0400, 1 July 2019.

## Results

**Figure 1:**
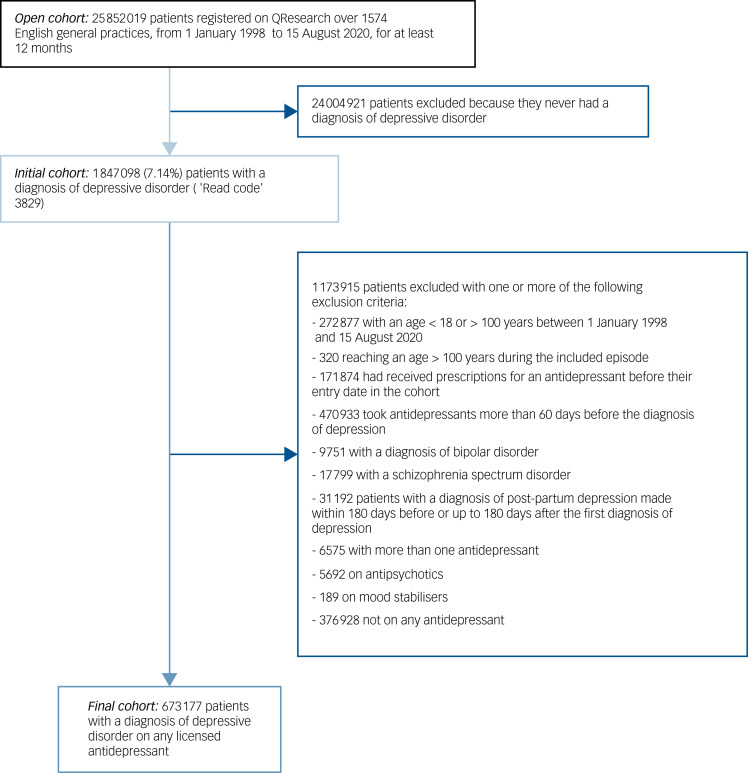
Flowchart of the study cohort.

From an open cohort of 25 852 019 people registered on QResearch over 1574 English general practices from 1 January 1998 to 15 August 2020 for at least 12 months, we identified an initial cohort of 1 847 098 participants (7.1%) with a first incident diagnosis of depressive disorder. Following the application of exclusion criteria, we obtained a final cohort of 673 177 people with a diagnosis of depressive disorder on any licensed antidepressant monotherapy. Of note, we excluded from the analysis 376 928 people who were not on antidepressants, but they were included in the multiple imputation process as they all carry information about missing values. The flowchart of the study cohort is shown in Fig. 1.

Baseline characteristics of the study population (*N* = 673 177) are in Table 1. Most participants were females (57.1%), with a mean age of 42.8 years (s.d. 17.7). Mean PHQ-9 score at baseline was 17.1 (s.d. 5.0), consistent with moderately severe depression.^20^

**Table 1:** Baseline characteristics of the study population. Values are numbers (percentages) unless stated otherwise

Baseline characteristics	Study population *N* = 673 177
Gender	
Female	384 432 (57.1)
Male	288 745 (42.9)
Mean age [s.d.]	42.75 [17.69]
Ethnic group	*N* = 493 323
Bangladeshi	5596 (1.1)
Black African	8355 (1.7)
Caribbean	6003 (1.2)
Chinese	2069 (0.4)
Indian	7901 (1.6)
Other	18 072 (3.7)
Other Asian	7205 (1.5)
Pakistani	8261 (1.7)
White	429 861 (87.1)
Townsend deprivation score, in fifths	*N* = 670 888
1 (least deprived)	138 850 (20.7)
2	142 751 (21.3)
3	140 770 (21)
4	129 627 (19.3)
5 (most deprived)	118 890 (17.7)
Region of England	
East Midlands	24 190 (3.6)
East of England	25 228 (3.8)
London	139 046 (20.7)
North-East	23 395 (3.5)
North-West	134 057 (19.9)
South-Central	85 774 (12.7)
South-East	66 521 (9.9)
South-West	75 750 (11.3)
West Midlands	65 910 (9.8)
Yorkshire & the Humber	33 306 (5)
Body Mass Index [s.d.]	*N* = 524 120
	26.24 [5.93]
Alcohol (daily units)	*N* = 340 117
Non-drinker/trivial (<1)	186 656 (54.9)
Light (1–2)	112 452 (33.1)
Medium (3–6)	30 726 (9)
Heavy (7–9)	4692 (1.4)
Very heavy (>9)	5591 (1.6)
Smoking (cigarettes/day)	*N* = 620 297
Non-smoker	299 939 (48.3)
Ex-smoker	117 618 (19)
Light smoker (1–9)	186 010 (30)
Moderate smoker (10–19)	11 774 (1.9)
Heavy smoker (≥20)	4956 (0.8)
‘Read code’ for depression	
Major depression	458 055 (68)
Minor depression	193 984 (28.8)
Other	21 138 (3.1)
Year of diagnosis	
1998 to 2005	197 840 (29.4)
2006 to 2010	139 417 (20.7)
2011 to 2015	159 556 (23.7)
2016 to 2020	176 364 (26.2)
Patient Health Questionnaire (PHQ)-9, at baseline [s.d.]	*N* = 144 347
	17.09 [4.95]
Comorbidities, at baseline	
Anxiety	78 914 (11.7)
Arthritis	44 048 (6.5)
Asthma	101 689 (15.1)
Cancer	49 022 (7.3)
Coronary heart disease	23 098 (3.4)
Diabetes	29 663 (4.4)
Epilepsy/seizure	8864 (1.3)
Hypothyroidism	20 868 (3.1)
Liver failure	5247 (0.8)
Migraine	39 698 (5.9)
Osteoporosis	7848 (1.2)
Renal failure	2142 (0.3)
Stroke/transient ischaemic attack	17 348 (2.6)
Suicidality/self-harm	17 484 (2.6)
Use of other drugs, at baseline	
Anticoagulants	8015 (1.2)
Anticonvulsants	10 470 (1.6)
Antihypertensives	51 036 (7.6)
Aspirin	28 875 (4.3)
Bisphosphonates	2309 (0.3)
Contraceptives	39 673 (5.9)
Hypnotics	52 177 (7.8)
Statins	46 842 (7)

Exposures for people starting an antidepressant are in Table 2. Most were prescribed SSRIs (85.7%), while a minority were prescribed other antidepressants (7.4%) and tricyclic antidepressants (TCAs) (6.9%), and only few were on monoamine oxidase inhibitors (MAOIs) (0.01%). The four antidepressants most prescribed, i.e. citalopram (36.1%), fluoxetine (22.3%), sertraline (20.2%) and mirtazapine (5.8%) accounted for 84.4% of total prescriptions.

**Table 2:** Antidepressants exposure of the study population, divided by antidepressant category. Values are numbers (percentages) unless stated otherwise

Antidepressants exposure	Study population *N* = 673 177 (%)
TCAs		46 580 (6.9)
	Amitriptyline	21 528 (3.2)
	Clomipramine	636 (0.1)
	Dosulepin	16 187 (2.4)
	Doxepin	304 (0.1)
	Imipramine	802 (0.1)
	Lofepramine	6257 (0.9)
	Maprotiline	14 (0.0)
	Mianserin	65 (0.0)
	Nortriptyline	408 (0.1)
	Trimipramine	378 (0.1)
SSRIs		576 936 (85.7)
	Citalopram	242 930 (36.1)
	Escitalopram	19 026 (2.8)
	Fluoxetine	150 274 (22.3)
	Fluvoxamine	153 (0.0)
	Paroxetine	28 613 (4.3)
	Sertraline	135 940 (20.2)
MAOIs		85 (0.0)
	Moclobemide	66 (0.0)
	Phenelzine	10 (0.0)
Other antidepressants		49 576 (7.4)
	Agomelatine	30 (0.0)
	Duloxetine	1213 (0.2)
	Mirtazapine	39 253 (5.8)
	Nefazodone	238 (0.0)
	Reboxetine	337 (0.1)
	Trazodone	3835 (0.6)
	Venlafaxine	4623 (0.7)
	Vortioxetine	45 (0.0)
	Others^a^	12 (0.0)

MAOIs, monoamine oxidase inhibitors; SSRIs, selective serotonin reuptake inhibitors; TCAs, tricyclic antidepressants.

a. Amoxapine, isocarboaxazid, tranylcypromine and tryptophan had fewer than 5 participants and were therefore excluded from the table to comply with QResearch data management guidelines.

Study outcomes are divided between short term (i.e. 2 months) and long term (i.e. 12 months), and descriptive statistics for them are reported in Table 3.

**Table 3:** Descriptive statistics of outcomes at 2 months (short term) and at 12 months (long term)

Outcome	Short-term outcome – 2 months *N* = 673 177	Long-term outcome – 12 months *N* = 673 177
Acceptability			
Treatment accepted	413 442 (61.4%)		82 524 (12.3%)
Treatment not accepted	259 735 (38.6%)		590 653 (87.7%)
Tolerability			
Treatment tolerated	635 417 (94.4%)		589 321 (87.5%)
Treatment non-tolerated	37 760 (5.6%)		83 856 (12.5%)
Safety			
At least one adverse event	306 655 (45.6%)		479 039 (71.2%)
Death	4082 (0.6%)		17 380 (2.6%)
	Baseline *N* = 144 347	Short-term outcome – 2 months *N* = 72 457	Long-term outcome – 12 months *N* = 11 764
Efficacy – Patient Health Questionnaire (PHQ)-9 mean [s.d.]	17.1 [5.0]	12.3 [6.5]	12.9 [6.8]

### Short-term outcomes – 2 months

At 2 months, antidepressant treatment was discontinued by 259 735 individuals (38.6%) because of any cause, of which 37 760 (5.6%) were because of an adverse event. In terms of safety, the number of people experiencing at least one adverse event was 306 655 (45.6%), and 4082 (0.6%) individuals died. For effectiveness, PHQ-9 scores decreased from ‘moderately severe’ (17.1, s.d. 5.0) to ‘moderate’ (12.3, s.d. 6.5) – Table 3.

Adjusted analyses comparing any antidepressant against fluoxetine at 2 months are in Table 4. Fluoxetine was more acceptable than all TCAs, trimipramine, paroxetine, duloxetine, venlafaxine, mirtazapine, reboxetine and trazodone. The aORs ranged between 1.09 (99% CI 1.05 to 1.14) for paroxetine and 3.04 (99% CI 1.08 to 8.51) for mianserin. Only citalopram was associated with fewer dropouts than fluoxetine (aOR 0.95, 99% CI 0.92 to 0.97).

**Table 4:** Adjusted^a^ analyses comparing any antidepressant with fluoxetine at 2 months (short term) and 12 months (long term)

	Acceptability, at 2 months	Acceptability, at 12 months	Tolerability, at 2 months	Tolerability, at 12 months	Safety, at 2 months	Safety, at 12 months	Response 50% reduction on PHQ-9, at 2 months	Response, 50% reduction on PHQ-9, at 12 months	Remission, scoring < 5 on PHQ-9, at 2 months	Remission, scoring < 5 on PHQ-9, at 12 months
Antidepressant	*N* = 673 176 aOR^a^ (99% CI)	*N* = 673 155 aOR^a^ (99% CI)	*N* = 673 160 aOR^a^ (99% CI)	*N* = 673 170 aOR^a^ (99% CI)	*N* = 673 174 aOR^a^ (99% CI)	*N* = 673 176 aOR^a^ (99% CI)	*N* = 669 679 aOR^a^ (99% CI)	*N* = 669 679 aOR^a^ (99% CI)	*N* = 669 679 aOR^a^ (99% CI)	*N* = 669 679 aOR^a^ (99% CI)
Fluoxetine *(index)*	1.00	1.00	1.00	1.00	1.00	1.00	1.00	1.00	1.00	1.00
Amitriptyline	2.92 (2.75, 3.09)	2.27 (2.06, 2.50)	3.36 (3.12, 3.63)	1.86 (1.75, 1.98)	1.55 (1.46, 1.65)	1.42 (1.33, 1.51)	0.39 (0.30, 0.50)	0.78 (0.61, 1.02)	0.42 (0.33, 0.54)	0.80 (0.60, 1.07)
Clomipramine	1.54 (1.26, 1.89)	1.11 (0.74, 1.67)	1.92 (1.31, 2.81)	1.30 (0.94, 1.8)	1.24 (0.99, 1.55)	1.09 (0.85, 1.39)	−	−	−	−
Dosulepin	1.35 (1.27, 1.43)	1.29 (1.17, 1.41)	1.48 (1.35, 1.63)	1.18 (1.09, 1.27)	1.12 (1.03, 1.21)	1.10 (1.01, 1.19)	0.71 (0.55, 0.91)	1.02 (0.61, 1.69)	0.73 (0.57, 0.93)	1.05 (0.64, 1.74)
Doxepin	1.60 (1.13, 2.25)	1.23 (0.81, 1.88)	1.94 (1.09, 3.46)	1.28 (0.76, 2.16)	1.04 (0.69, 1.55)	1.07 (0.77, 1.50)	−	−	−	−
Imipramine	1.58 (1.24, 2.02)	1.27 (0.90, 1.80)	1.92 (1.29, 2.88)	1.51 (1.15, 1.97)	1.05 (0.83, 1.33)	1.13 (0.91, 1.40)	−	−	−	−
Lofepramine	1.62 (1.50, 1.76)	2.06 (1.77, 2.41)	1.64 (1.44, 1.87)	1.27 (1.14, 1.41)	1.15 (1.04, 1.28)	1.11 (1.00, 1.23)	0.70 (0.48, 1.03)	0.64 (0.27, 1.56)	0.72 (0.51, 1.01)	0.64 (0.26, 1.58)
Maprotiline	2.09 (0.37, 11.9)	1	4.62 (1.16, 18.5)	2.64 (0.84, 8.28)	1.40 (0.25, 7.93)	1.35 (0.21, 8.51)	−	−	−	−
Mianserin	3.04 (1.08, 8.51)	9.34 (0.65, 133)	2.13 (0.72, 6.28)	1.20 (0.49, 2.92)	0.96 (0.52, 1.76)	1.21 (0.62, 2.35)	−	−	−	−
Nortriptyline	2.50 (1.83, 3.41)	2.20 (1.27, 3.80)	3.66 (2.62, 5.12)	2.46 (1.80, 3.35)	1.56 (1.19, 2.06)	1.28 (0.93, 1.77)	−	−	−	−
Trimipramine	1.60 (1.15, 2.23)	1.52 (0.84, 2.76)	2.26 (1.37, 3.72)	1.48 (1.01, 2.19)	1.00 (0.71, 1.42)	1.07 (0.77, 1.50)	−	−	−	−
Moclobemide	0.98 (0.52, 1.86)	0.92 (0.33, 2.59)	0.33 (0.03, 4.02)	1.80 (0.80, 4.04)	0.88 (0.46, 1.71)	0.87 (0.47, 1.62)	−	−	−	−
Phenelzine	1.70 (0.32, 8.98)	0.47 (0.07, 3.30)	1	0.93 (0.06, 14.2)	1.31(0.24, 7.22)	0.76 (0.15, 3.88)	−	−	−	−
Citalopram	0.95 (0.92, 0.97)	0.78 (0.76, 0.81)	1.03 (0.99, 1.08)	1.09 (1.06, 1.13)	1.17 (1.12, 1.21)	1.12 (1.08, 1.16)	1.04 (0.98, 1.10)	1.12 (1.01, 1.23)	1.04 (0.98, 1.10)	1.12 (1.01, 1.25)
Escitalopram	0.97 (0.91, 1.02)	0.83 (0.78, 0.90)	1.04 (0.94, 1.15)	1.07 (0.99, 1.16)	1.15 (1.05, 1.26)	1.07 (0.97, 1.19)	0.88 (0.75, 1.04)	1.07 (0.77, 1.48)	0.88 (0.74, 1.05)	1.08 (0.77, 1.53)
Fluvoxamine	1.18 (0.74, 1.87)	1.12 (0.53, 2.39)	1.89 (0.86, 4.18)	1.32 (0.71, 2.46)	0.80 (0.47, 1.34)	0.73 (0.37, 1.46)	−	−	−	−
Paroxetine	1.09 (1.05, 1.14)	0.84 (0.79, 0.90)	1.18 (1.08, 1.28)	1.07 (1.01, 1.14)	1.11 (1.04, 1.18)	1.01 (0.94, 1.07)	0.83 (0.68, 1.02)	0.98 (0.66, 1.44)	0.86 (0.69, 1.07)	1.00 (0.69, 1.45)
Sertraline	1.00 (0.97, 1.03)	0.84 (0.81, 0.87)	1.12 (1.06, 1.18)	1.11 (1.07, 1.15)	1.25 (1.20, 1.31)	1.14 (1.09, 1.19)	1.02 (0.93, 1.11)	1.15 (0.93, 1.42)	1.02 (0.93, 1.12)	1.15 (0.92, 1.43)
Agomelatine	1.56 (0.60, 4.07)	2.12 (0.29, 15.5)	1.46 (0.22, 9.66)	0.94 (0.20, 4.39)	1.17 (0.47, 2.91)	0.73 (0.26, 2.04)	−	−	−	−
Duloxetine	1.24 (1.06, 1.46)	0.93 (0.75, 1.15)	1.59 (1.20, 2.12)	1.61 (1.33, 1.96)	1.27 (1.08, 1.50)	1.21 (0.99, 1.48)	0.79 (0.51, 1.23)	0.41 (0.06, 2.73)	0.80 (0.53, 1.2)	0.39 (0.05, 3.08)
Mirtazapine	1.35 (1.31, 1.41)	1.22 (1.16, 1.29)	1.40 (1.30, 1.49)	1.21 (1.15, 1.27)	1.21 (1.15, 1.27)	1.14 (1.08, 1.19)	0.78 (0.71, 0.87)	0.92 (0.75, 1.14)	0.79 (0.71, 0.88)	0.93 (0.73, 1.18)
Nefazodone	1.16 (0.81, 1.66)	0.93 (0.55, 1.57)	1.17 (0.52, 2.63)	1.20 (0.67, 2.17)	0.74 (0.48, 1.12)	0.98 (0.67, 1.42)	−	−	−	−
Reboxetine	2.07 (1.16, 3.67)	1.77 (0.76, 4.09)	1.92 (1.09, 3.39)	1.30 (0.89, 1.89)	1.00 (0.73, 1.36)	0.97 (0.70, 1.35)	−	−	−	−
Trazodone	1.74 (1.27, 2.40)	1.52 (1.21, 1.90)	1.99 (1.59, 2.49)	1.42 (1.25, 1.61)	1.37 (1.19, 1.58)	1.26 (1.08, 1.48)	0.62 (0.47, 0.83)	0.73 (0.35, 1.52)	0.63 (0.43, 0.93)	0.75 (0.36, 1.58)
Venlafaxine	1.45 (1.33, 1.57)	1.13 (0.98, 1.29)	1.57 (1.35, 1.84)	1.30 (1.16, 1.46)	1.09 (1.00, 1.19)	1.04 (0.94, 1.14)	0.79 (0.52, 1.17)	0.76 (0.39, 1.45)	0.80 (0.55, 1.16)	0.76 (0.37, 1.58)
Vortioxetine	1.08 (0.48, 2.42)	1.13 (0.28, 4.52)	0.48 (0.04, 6.52)	0.87 (0.23, 3.36)	0.77 (0.35, 1.69)	0.66 (0.30, 1.46)	−	−	−	−

a. The numbers in each cell correspond to the estimated adjusted odds ratios (aORs) for each outcome and antidepressant treatment compared to fluoxetine. This was adjusted for clusters in general practices and for gender, age, baseline severity, depression subtype, year of diagnosis, body mass index (BMI), smoking status, daily alcohol intake, ethnicity, Townsend quintile, region of England, comorbidities at baseline (coronary heart disease, stroke/transient ischaemic attack, diabetes, cancer, epilepsy/seizures, hypothyroidism, osteoarthritis/rheumatoid arthritis, suicidal ideation/behaviour or self-harm, asthma/chronic obstructive pulmonary disease [COPD] , osteoporosis, liver disease, renal disease, anxiety) and use of other drugs at baseline (anticonvulsants, hypnotics/anxiolytics, antihypertensive drugs, aspirin, statins, anticoagulants, non-steroidal anti-inflammatory drugs, bisphosphonates, oral contraceptives/hormone replacement therapy). Amoxapine, isocarboaxazid, tranylcypromine and tryptophan, which had fewer than five patients, were excluded from the table to comply with QResearch data management guidelines. The shading corresponds to the strength of statistical evidence regarding the effects versus fluoxetine. A dark cell with background lines indicates strong evidence (*P* < 0.001) that the corresponding drug performs better than fluoxetine for the corresponding outcome. Conversely, a dark cell without lines indicates strong evidence (*P* < 0.001) that the drug performs worse than fluoxetine. Colours closer to white indicate lack of evidence on whether the drug performs better or worse than fluoxetine (see legend below).

**Legend** 
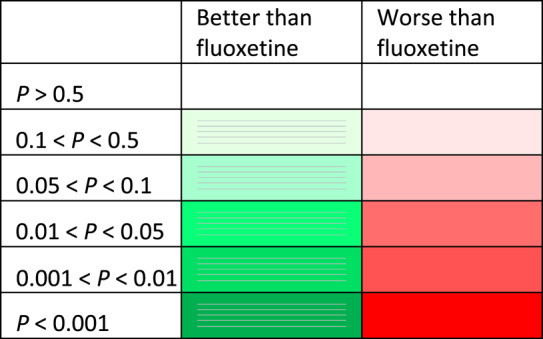

Fluoxetine was more tolerable than all TCAs but mianserin (aOR 2.13, 99% CI 0.72 to 6.28), and also more tolerable than trimipramine, paroxetine, sertraline, duloxetine, venlafaxine, mirtazapine, reboxetine and trazodone. The aORs ranged between 1.12 (99% CI 1.06 to 1.18) for sertraline and 4.62, (99% CI 1.16 to 18.46) for maprotiline.

Fluoxetine was found to be safer than amitriptyline, dosulepin, lofepramine and nortriptyline among the TCAs, as well as mirtazapine, trazodone, venlafaxine and duloxetine. Further, fluoxetine was found to be the safest of all SSRIs except for fluvoxamine, for which less information was available (aOR 0.80, 99% CI 0.47 to 1.34).

For effectiveness, amitriptyline, dosulepin, trazodone and mirtazapine were less efficacious in terms of response and remission than fluoxetine, with aORs ranging from 0.39 (99% CI 0.30 to 0.50) and 0.42 (99% CI 0.33 to 0.54) for amitriptyline, and 0.78 (99% CI 0.71 to 0.89) and 0.79 (99% CI 0.71 to 0.88) for mirtazapine. No drug was found to be more efficacious.

### Long-term outcomes – 12 months

At 12 months, antidepressant treatment was discontinued by 590 653 individuals (87.7%), of which 83 856 (12.5%) were because of adverse events. In terms of safety, the number of people experiencing at least one side-effect was again high at 479 039 (71.2%), and all-cause mortality was recorded for 14 172 (2.1%) people. For effectiveness, PHQ-9 scores decreased from ‘moderately severe’ at baseline (17.1, s.d. 5.0) to ‘moderate’ (12.9, s.d. 6.8) – Table 3.

Adjusted analyses comparing any antidepressant against fluoxetine at 12 months are in Table 4.

Fluoxetine was more acceptable than amitriptyline, dosulepin, lofepramine, nortriptyline, mirtazapine and trazodone, with aORs ranging between 2.27 (99% CI 2.06 to 2.50) for amitriptyline and 1.22 (99% CI 1.16 to 1.29) for mirtazapine. Four antidepressants were more acceptable than fluoxetine at 12 months: citalopram (aOR 0.78, 99% CI 0.76 to 0.81), escitalopram (aOR 0.83, 99% CI 0.78 to 0.90), paroxetine (aOR 0.84, 99% CI 0.79 to 0.90) and sertraline (aOR 0.84, 99% CI 0.79 to 0.90).

Fluoxetine was more tolerable than amitriptyline, dosulepin, imipramine, lofepramine and nortriptyline among the TCAs, as well as trimipramine, duloxetine, venlafaxine, mirtazapine and trazodone. All SSRIs but escitalopram and fluvoxamine were less tolerable than fluoxetine. The aORs ranged between 2.46 (99% CI 1.80 to 3.35) for nortriptyline and 1.07 (99% CI 1.01 to 1.14) for paroxetine.

Fluoxetine was safer than amitriptyline, dosulepin, citalopram, sertraline, mirtazapine and trazodone with aORs ranging between 1.42 (99% CI 1.33 to 1.51) for amitriptyline and 1.10 (99% CI 1.01 to 1.19) for dosulepin.

For effectiveness, we only found evidence that citalopram was more efficacious than fluoxetine for both response (aOR 1.12, 99% CI 1.01 to 1.23) and remission (aOR 1.12, 99% CI 1.01 to 1.25).

### Comparison of real-world versus RCTs data

**Figure 2:**
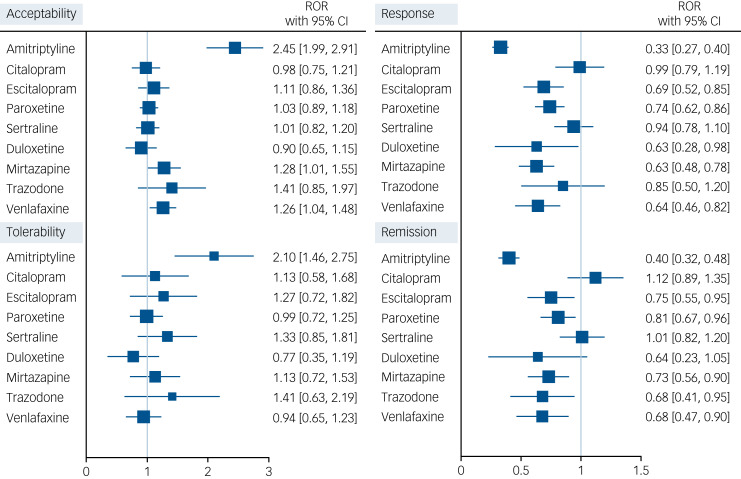
Forest plot showing ratio-of-odds ratios (ROR) with 95% CI for each antidepressant, with fluoxetine as index comparator, comparing real-world versus clinical trials (Group of Researchers Investigating Specific Efficacy of Individual Drugs for Acute Depression [GRISELDA] study) data. For acceptability and tolerability, a ROR >1 for drug X indicates that the effect of X versus fluoxetine is larger in randomised controlled trials (RCTs) as compared with the real world. For response and remission, a ROR <1 for drug X indicates that the effect of X versus fluoxetine is larger in RCTs as compared with the real world.

We calculated RORs with 95% CI estimated at 2 months for each antidepressant (fluoxetine as index comparator), comparing real-world with randomised data in Fig. 2. For acceptability, amitriptyline, mirtazapine and venlafaxine fared better in RCTs than in real-world data, with RORs ranging from 1.26 (95% CI 1.06 to 1.49) for venlafaxine to 2.45 (95% CI 2.03 to 2.95) for amitriptyline. Amitriptyline also showed to be better tolerated in RCTs than in the real world (ROR 2.10, 95% CI 1.55 to 2.84). For response and remission, amitriptyline, escitalopram, paroxetine, mirtazapine and venlafaxine were superior in RCTs, with RORs ranging from 0.33 (95% CI 0.27 to 0.40) and 0.40 (95% CI 0.32 to 0.49) for amitriptyline to 0.74 (95% CI 0.63 to 0.87) and 0.81 (95% CI 0.68 to 0.97) for paroxetine.

## Discussion

This study investigated the comparative effects of antidepressants in a cohort of 673 177 participants with a diagnosis of depressive disorder, in a large community-based sample from UK primary care, followed up for 12 months in real-world conditions. The size and detail of available data allowed us to control for numerous potential confounders and to assess several outcomes, including antidepressant acceptability, tolerability, safety (overall and for individual adverse events) and effectiveness (response and remission).

In terms of acceptability, antidepressants dropout rates were high (38.6% at 2 months, 87.7% at 12 months). In RCTs, based on a network meta-analysis of 522 trials, 26% of people on average discontinued antidepressants because of any cause at 2 months.^9^ In the Sequenced Treatment Alternatives to Relieve Depression (STAR*D) trial, discontinuation rate in the short term was 27%.^10^ Some studies performed in similar naturalistic settings are in line with our results, with discontinuation rates around 43% at 6 weeks and 73% at 6 months,^25-28^ although other epidemiology surveys have reported much lower rates.^29^ It is also possible that some of the recorded discontinuations from antidepressant treatment would have occurred in individuals whose depressive symptoms had resolved – such could occur in both clinical trials (e.g. individuals who are lost to follow-up) and observational studies. In our study, this occurrence might also be a consequence of including people with diagnostic codes for ‘minor depression’, as done in similar previous studies on QResearch.^16,17^ However, this should not be a major issue in this study of primary care patients, because clinical guidelines followed by general practitioners (GPs) in the UK advise continuing antidepressant medication for at least six months after symptomatic remission.^7^ The fact that antidepressants dropout rates were higher in real-world data than in trials could be because of a variety of factors. In naturalistic settings, people are often prescribed medication with limited focus on ensuring adherence with treatment; conversely, RCTs tend to enrol participants who are highly motivated to comply with medication,^30^ which might contribute to lower external validity of trial results. In the routine clinical practice world, high discontinuation rates of antidepressants can have serious consequences for people, as they are associated with a high risk of relapse.^31^ The difficulty of accessing treatment is another significant factor contributing to the high discontinuation rates of antidepressants. In the UK National Health System, access to antidepressant medications through general practitioners can be challenging for some individuals because of limited consultation time and stigma, as well as prescription restrictions, both due to reimbursement constraints and primary care guidelines that limit the choice of antidepressants.^32^ Our observed low acceptability for antidepressants therefore suggests the importance of improving shared decision-making between patients and healthcare professionals to increase access to appropriate antidepressant treatments and reduce discontinuation rates in routine clinical practice.^33^

Antidepressant treatment was stopped because of adverse events in a non-negligible proportion of participants (5.6% at 2 months, 12.5% at 12 months). Most suffered at least one adverse event (45.6% at 2 months, 71.2% at 12 months), and 2.1% of them died during the 12-month follow-up. Our findings on the tolerability and safety of antidepressants complement the body of research carried out to date using QResearch. Previous work has focused on the association between use of antidepressants and important adverse events such as falls, fractures, upper gastrointestinal bleed, road traffic accidents, adverse drug reactions, all-cause mortality,^17^ epilepsy and seizure,^34^ myocardial infarction, stroke or transient ischaemic attack, arrhythmia,^35^ and suicide and attempted suicide or self-harm,^36^ as well as referral for an adverse event in older people.^16^ Nevertheless, adverse events appeared to be a minor reason for discontinuation, which is in line with RCT data where 10% of people discontinue antidepressants due to any adverse event (i.e. tolerability) within 2 months.^9^

For effectiveness, a reduction in the PHQ-9 score was observed, with average depression severity decreasing from ‘moderately severe’ at baseline to ‘moderate’ at 2 months and 12 months. There was an overall improvement, but moderate depressive symptoms were still present at both 2 months and 12 months. This result is in line with the modest effect sizes for antidepressants seen in RCTs.^9^

We further assessed the credibility of our findings obtained using real-world data by comparing them with results from a large data-set of clinical trials, GRISELDA. Clinical trials are the most reliable source of information when comparing relative treatment effects,^37^ but their rigorous experimental setting and restrictive inclusion/exclusion criteria may potentially limit their generalisability to clinical practice.^12^ For example, RCTs often exclude individuals with chronic multimorbidity and those who are taking multiple medications due to the need to control for variables that could affect study outcomes.^11^ If such characteristics are strong effect modifiers, i.e. if they modify relative treatment effects, then the transportability of RCT findings to real-world settings may be jeopardised. On this basis, the comparison of the findings from our study with those of the clinical trials data-set GRISELDA supports our conclusions while also providing novel insights. In our analyses, we found that several of the most used antidepressants (i.e. trazodone, citalopram, escitalopram, paroxetine, sertraline and duloxetine, compared with fluoxetine) showed a similar effect size in both data-sets, with citalopram and sertraline displaying very similar results in the real world and in RCTs. However, amitriptyline, mirtazapine and venlafaxine showed a far better profile in the short-term treatment of depression in RCTs compared with real-world data, where they fared poorly. These conflicting findings could be due to several factors, including selective inclusion and exclusion criteria in RCTs, variable placebo effect observed in antidepressant trials,^38^ unmeasured confounding in real-world studies, missing outcomes being not random in the real world, and the low number of RCTs directly comparing these drugs in GRISELDA.^9^ Furthermore, it should be noted that our cohort study included individuals using first-line antidepressant monotherapy, while amitriptyline, mirtazapine and venlafaxine are more commonly used in primary care as second-line treatments, usually after one or more courses of SSRIs. Nevertheless, the overlap of effect sizes between observational and randomised data strengthens the available body of evidence on antidepressant effects. It also highlights how large, detailed and rigorous observational studies, which follow a methodology akin to clinical trials,^39^ can complement evidence provided by the latter while addressing some of their limitations. Future research should move from a population-based perspective, toward a personalised-based approach to tailor antidepressants to people's individual characteristics.

### Strengths and limitations

This study includes the largest and most detailed cohort of people with depressive disorder investigated under real-world conditions to assess the comparative effectiveness of antidepressant monotherapy. The use of an active index comparator (i.e. fluoxetine) fills an important evidence gap and has been previously advocated.^40^ Despite its observational nature, this study focuses on clinically relevant outcomes that are similarly evaluated in RCTs,^9^ while also including an in-depth evaluation of side effects and a longer follow-up (i.e. 12 months). Moreover, a representative cohort of adults with depression in England could be examined, including people with comorbidities and concomitant medication use, who are usually excluded from clinical trials. Including such individuals constitutes a significant added value of this study, whose results aim to be generalised to the wider population (i.e. high external validity). However, people with certain comorbid conditions such as cognitive impairment (though unlikely to be common for our sample with a mean age of ~42 years) may show responses to antidepressants that are substantially different from those of others.

The main limitation of this study involves a weaker internal validity compared with RCTs, primarily because of potential indication bias. RCTs that aim to estimate causal effects of interventions are mostly at lower risk of bias when compared with observational investigations.^41^ In our study, we attempted to minimise biases by only including individuals with a diagnosis of depression and by controlling for potentially confounding variables^16,17^ recorded in considerable detail on QResearch.^42^ Nevertheless, we cannot exclude the possibility of important differences in unmeasured effect modifiers between participants prescribed different antidepressant medications. Such differences would introduce bias to the associations between the drug exposures and the outcomes, and perhaps explain some of the observed differences with RCT data. Channelling bias, which occurs when treatments with similar indications are prescribed to different patient segments based on a different perceived prognosis, must also be considered.^43^ Although all participants in our sample had been newly diagnosed with depression, those with lower depressive symptoms scores on the PHQ-9 may have been treated differently from those with higher scores. Second, the effectiveness of some antidepressants could not be assessed in view of the low number of PHQ-9 scores available at baseline and at study endpoints for these drugs. The PHQ-9 is a validated self-rating scale, which is reliable for measuring depressive symptoms over time but not good for diagnosis.^20^ The availability of other depression scales more commonly employed in RCTs of antidepressants (e.g. Montgomery-Åsberg Depression Scale or Hamilton Depression Rating Scale) would allow better comparison and stronger consistency between real-world and RCTs data, but these observer-rating scales are not used in real-world practice.

Moreover, as pre-specified in the published protocol,^15^ we used 10 imputed datasets to account for missing data. This number of imputations is the same as previous studies using QResearch;^44,45^ however, we acknowledge that a larger number of imputations could have further increased the precision and the reproducibility of the result estimates.^46^ Third, we decided to dichotomise the PHQ-9 scores. The dichotomisation of a continuous variable leads to a potential loss of information. However, dichotomisation was required in our analysis to allow the comparison with the GRISELDA data-set. Fourth, the use of Read Codes for identification of adverse events may have resulted in data omission where such events were recorded in clinical encounter notes. Under-recording or miscoding of these data may have also occurred as this is a common issue with studies using routinely collected electronic health records; however, we have no reason to believe that this problem, if present, was differentiated by type of antidepressant. Fifthly, we decided not to employ a Cox proportional-hazard model for the analyses, as initially planned,^15^ because the proportional-hazard assumption for the outcomes measured was found to be implausible in our data. Instead, we employed multiple logistic regression models at pre-specified timepoints, as this analysis does not rely on proportional-hazard assumption, while also allowing comparison with previous clinical trials.^9^ Nevertheless, a time-to-event analysis would provide better information about varying lengths of follow-up; this might be pursued in future research.

Additionally, our data-set is based primarily on prescription orders rather than actual medication dispensing. Although such missing data could affect the magnitude of observed changes across the entire sample, they were unlikely to impact the comparative analysis of relative changes between individual antidepressants and fluoxetine.

Finally, we could not assess the dose of initiated antidepressants, meaning that some medications (e.g. amitriptyline) may have been prescribed below usual antidepressant dosage.

## Conclusion

Our study included a comprehensive analysis of real-world data from a cohort of 673 177 depressed individuals receiving first-line treatment with antidepressants in England. We found evidence of low acceptability, good tolerability and safety, and small-to-moderate effectiveness of antidepressants. SSRIs, including citalopram, fluoxetine and sertraline had the most favourable benefit/risk profile with good tolerability, safety and small-to-moderate effectiveness in both short- and long-term use. Additionally, our comparison of real-world estimates with data from RCTs showed good agreement for the most frequently prescribed antidepressants.

## Supporting information

De Crescenzo et al. supplementary material De Crescenzo et al. supplementary material

## Data Availability

The data that support the findings of this study are not publicly available because they are based on de-identified national clinical records. Because of national and organisational data privacy regulations, individual-level data such as those used for this study cannot be shared openly.
