# Supplementary material for: Real-world effects of antidepressants for depressive disorder in primary care: population-based cohort study
Source: Br J Psychiatry. 2024 Dec 5;226(5):278–87. doi: 10.1192/bjp.2024.194 (PMC12116218; doi:10.1192/bjp.2024.194)
Supplement: De Crescenzo et al. supplementary material [file S0007125024001946sup001.docx]

**Appendix 1** – Variables with missing data.

| **Variable** | **Missing** | **% Missing** | **Not Missing** | **Unique values** | **Min** | **Max** |
| --- | --- | --- | --- | --- | --- | --- |
| Ethnicity | 262,202 | 24.97 | 787,903 | 9 | 1 | 9 |
| BMI | 217,391 | 20.7 | 832,714 | >500 | 10 | 100 |
| Smoker | 75,281 | 7.17 | 974,824 | 5 | 0 | 4 |
| Townsend Quintiles | 3,505 | 0.33 | 1,046,600 | 5 | 0 | 4 |
| PHQ-9 Baseline | 853,344 | 81.26 | 196,761 | 28 | 0 | 27 |
| PHQ-9 - 2 months | 962,109 | 91.27 | 87,996 | 28 | 0 | 27 |
| PHQ-9 - 12 months | 1,033,225 | 98.39 | 16,880 | 27 | 1 | 27 |
